# What Determines the Probability of Discovering a Species? A Study of the Completeness of Bryophyte Inventories in Tianmushan National Nature Reserve (Zhejiang, China)

**DOI:** 10.1002/ece3.70593

**Published:** 2024-11-21

**Authors:** Xue Yao, Zun Dai, Yiran Wang, Jian Zhang, Jian Wang

**Affiliations:** ^1^ Bryology Laboratory, School of Life Sciences East China Normal University Shanghai China; ^2^ School of Life Sciences Sun Yat‐Sen University Guangzhou China; ^3^ Shanghai Institute of Eco‐Chongming (SIEC) Shanghai China

**Keywords:** bryophytes, completeness, newly recorded species, species diversity, species inventory, temporal turnover

## Abstract

Knowing the total number of species in a region has been a question of great interest motivated by the need to provide a reference point for current and future losses of biodiversity. Unfortunately, obtaining an accurate number is constrained by the fact that most species remain to be discovered, due to the imperfect detection of species in the field collection or because of temporal turnover in species composition. Here, to understand the inventory completeness at the local scale, we studied the temporal dynamics in the species richness and composition of bryophytes in Tianmushan National Nature Reserve. We used β‐diversity to measure the temporal variation and analyzed the species attributes of newly discovered species collected this time. Furthermore, we evaluated our sampling strategy to measure sampling effect and estimated the species that remain to be discovered. We found that total β‐diversity was largely driven by turnover. The analysis of species attributes showed that epiphytic species dominate the newly recorded species in both the narrow and wide elevational range species. Further, if only one of the methods was adopted, 26%–29% of the newly discovered species would be missed, and we inferred that there are likely are 185 bryophytes yet to be discovered. Our results indicate that when the same effort was made, an appropriate sampling methodology is crucial to accelerate newly recorded species discovery. Further, the results of our study highlight that species temporal turnover should be considered when assessing the completeness of species inventories at the local scale.

## Graphical abstract

Knowing the total number of species in a region has been a question of great interest motivated by the need to provide a reference point for current and future losses of biodiversity. Here, to understand the inventory completeness at the local scale, we studied the temporal dynamics in the species richness and composition of bryophytes in Tianmushan National Nature Reserve. Our results indicate that species temporal turnover should be considered when assessing the completeness of species inventories at the local scale.

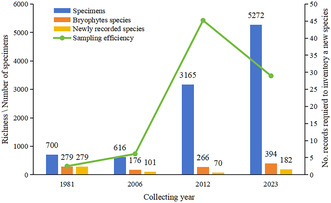

## 1 Introduction

Biodiversity loss is one of the worst impacts of human activities on Earth's ecosystems (Jones et al. 2018). One widely accepted response to this crisis is the establishment of protected areas (PAs) as a key policy tool for global biodiversity conservation (Geldmann, Manica, and Burgess 2019; Gatiso et al. 2022). When establishing priorities for conservation areas and nature reserve policies, it is crucial to have access to accurate information on species diversity and distribution (Aranda et al. 2010). Species inventories aim to document a variety of species within a specific location and timeframe, are routinely conducted, and provide valuable insights for assessing biodiversity and its changes (Guralnick, Walls, and Jetz 2018). Nonetheless, insufficient sampling can lead to plants being overlooked or underrepresented in field studies (Chen et al. 2013). Even for well‐studied groups like vascular plants and vertebrates, accurate records of their diversity and distribution remain lacking (Hurlbert and Jetz 2007; Costello, May, and Stork 2013).

Similar to the species‐area relationship, there exists a species‐time relationship (Preston 1960), where the longer an area is surveyed, the greater the number of species observed, albeit at a declining rate as time progresses due to reduced sampling efficiency (e.g., diminishing returns in terms of newly recorded species observed) (Korhonen, Soininen, and Hillebrand 2010). This is primarily because common species are quickly discovered and collected in the initial stages, while the discovery of rarer taxa becomes more challenging as the survey approaches completion (Aranda et al. 2010). Numerous studies have found that rare species often share common attributes, such as small body size, limited distribution range, and specific elevation preferences (Chen, Zheng, and Liu 2023, and references therein).

On the other hand, it is increasingly evident that changes in the composition of local assemblages (temporal turnover in composition) represent a significant signal of biodiversity changes in the face of climate change and extensive human influence (Dornelas et al. 2023). Since all assemblages undergo temporal turnover, the composition of the assemblage may become increasingly different from its previous state with the passage of time (Diamond and May 1977). While the mechanisms behind temporal turnover are not yet fully understood, over shorter time periods (ranging from weeks to years), the changes in assemblage composition are typically influenced by a combination of sampling effect and ecological factors, such as the arrival of newly recorded species and the disappearance of existing species due to changes in the environment (Preston 1960; Storch, Šizling, and Gaston 2007; Korhonen, Soininen, and Hillebrand 2010). Therefore, in order to gain a comprehensive understanding of the completeness of species inventory at a local scale, it is essential to examine the varying impacts of these two factors on changes in assemblage composition over time. To the best of our knowledge, there have been limited studies on bryophytes that have specifically explored this issue.

Thanks to the pioneering efforts of many bryologists, the bryoflora described for Tianmushan National Nature Reserve (TNNR, Zhejiang, China) is well known. Since 1977, there have been regular inventories of bryophytes in TNNR, leading to several published studies (Hu and Wang 1981; Wang et al. 2012; Chen et al. 2023) that have provided comprehensive lists of species. The initial species catalog for TNNR was released in 1981, documenting a total of 279 species (Hu and Wang 1981). This catalog has been updated by Li et al. (2006), Wang et al. (2012), and Wang et al. (2013), which added 101, 23, and 47 newly recorded species to the recorded inventory, respectively. The analysis of newly recorded species over time in TNNR suggests that the discovery of newly recorded species is becoming more challenging as the species inventory becomes more extensive. However, during a recent field work in TNNR, we identified 182 newly recorded species, supplementing the existing pool of observed species (Chen et al. 2023). These results indicate that the current sampling strategies may not sufficiently capture the full biodiversity of TNNR. Conversely, they may also suggest potential shifts in the composition of species assemblages over time.

In this study, utilizing an extensive bryophyte dataset in TNNR, we aim to assess the completeness of species inventories and attempt to answer the question of what led to the discovery of so many newly recorded species by Chen et al. (2023). Specifically, our aims are to: (1) outline the historical progression of bryophyte cataloging and the temporal patterns in the cataloging of newly documented species, followed by evaluating the effectiveness of sampling (the ratio between the number of database‐records and the number of new inventoried species) over time; (2) measure the extent of compositional change in assemblages over time; (3) examine species attributes (elevation range and habitat preferences) of all 182 newly inventoried species; (4) evaluate whether our sampling strategy (e.g., sampling method, sampling effort) is more conductive to discover more new recorded species; and (5) determine the quantity of species not yet found in TNNR and identify potential range for discovery.

## 2 Materials and Methods

### 2.1 Study Sites

Tianmushan National Nature Reserve, covering 42.84 km^2^, is located in the northerly part of the mid‐subtropical zone in the Yangtze River Delta region, one of China's most populated and rapidly developing areas. Located between 119°23′47″–119°28′27″ latitude and 30°18′30″–30°24′55″ longitude, it is part of the International Biosphere Reserve (MAB) network. The reserve experiences a wet monsoon climate, with annual temperatures ranging from 8.8°C to 14.8°C and annual precipitation averaging between 1390 and 1870 mm. The highest rainfall is typically observed at elevations of 900–1000 m (Tianmushan Nature Reserve Administration 1992). Due to its rugged topography and significant climatic shifts based on elevation, Mt. Tianmu displays distinct vegetation zones: (1) evergreen broad‐leaved forest zone (< 950 m), (2) evergreen and deciduous broad‐leaved mix forest zone (950–1200 m), and (3) deciduous broad‐leaved forest zone (1200–1506 m) (Shang, Chen, and Da 2018).

### 2.2 Biological Database

We compiled all the known information on bryophyte distribution of TNNR till now and then digitized them following a standard protocol. After examining the specimens preserved at the Herbarium of the East China Normal University (HSNU), including a high proportion of our recent collections, we obtained the following collection information for each species: year of collection, collector, locality, altitude, and type of substratum. We standardized the nomenclature according to Söderström et al. (2016) and Jia and He (2013). A total of 9753 records belonging to 638 species of 217 genera in 78 families were included in this study. We combined the databases of 2012 and 2013 due to the short inventory time intervals and the high overlapping species among these two databases. The inventory records of each year are presented in Table S2.

### 2.3 Assessment of Temporal Trends

The temporal trends of taxonomic inventories were described for each year of collection using all the available records from 1981 to 2023 in TNNR. We then analyzed historical trends in the inventorying of species by depicting the cumulative number of species and new recorded species that were added to the pool of all previously collected species over time. Using the number of database records as a surrogate for survey effort, we also estimated the sampling efficiency by calculating the number of database records that were necessary to discover a species previously unknown to TNNR.

### 2.4 Measuring Temporal Turnover in Species Composition

β‐diversity can provide a good understanding of the distribution patterns and driving mechanisms of various biological groups in different spatial and temporal dimensions (Si et al. 2017). According to Baselga (2010), the β‐diversity can be decomposed into two components: turnover and nestedness. A high turnover indicates a high rate of species replacement between sites, whereas a high nestedness rate underlies differences in species richness among sites (i.e., a low‐richness site is a subset of a high‐richness site). Such decomposition can help capture the underlying ecological processes that regulate community assembly (Baselga and Leprieur 2015; Wu et al. 2021). We used pairwise dissimilarity matrices to measure the temporal variation in species composition. To examine how the assemblage similarity changes with time, we calculated the Jaccard dissimilarity (β_jac_) and partitioned it into its turnover (β_jtu_) and nestedness (β_jne_) components using the beta.div.comp function in R package adespatial (Dray et al. 2023).

### 2.5 Examining Species Attributes of 182 Newly Discovered Species

Species attributes, such as body size, range size, and habitat breadth, have been found to have significant effects on the likelihood of species being encountered by collectors (Gaston, Blackburn, and Loder 1995; Essl et al. 2013; Colli et al. 2016). Early discovered species are more likely to be generalist species, which have a larger range size, a larger elevation range, and a larger body size (Chen, Zheng, and Liu 2023). To explore the relative importance of species attributes on species discovered probability, we compiled variables that are potentially associated with species discovered probability for each of 182 newly discovered species by Chen et al. (2023), including elevation range and body size. For elevation range, following Vanderpoorten and Engels (2003), species occurring in < 10% of the plots were considered as low frequency. As compared to vascular plants, the body size of bryophytes is relatively small and may be a weak predictor for their species discovery in the field. Therefore, we only explored the body size of the dominant groups of the 182 newly recorded species, aiming to identify discernible patterns within higher‐level taxonomic units. Considering that the ability of bryophytes to track areas of suitable climate depends on their habitat preferences (Dai, Zhang, and Wang 2023), we also analyzed habitat preferences for 182 species to assess the impact of climate change on these newly discovered species.

### 2.6 Evaluating Our Sampling Strategy and Inventory Completeness

An appropriate sampling methodology is fundamental to comprehending patterns of community and taxon diversity across the landscape, as highlighted by Vanderpoorten, Papp, and Gradstein (2010). To obtain a better inventory of bryophytes, both plot sampling (PS) and floristic habitat sampling (FHS) (Ilić et al. 2018) were used by Chen et al. (2023). In the subtropical forests of eastern China, the ground bryophytes exhibited a patchy distribution, often leading to variances in the number of plots without bryophytes. Consequently, we decided to conduct plot sampling exclusively for epiphytic bryophytes. In this study, thirty‐seven plots, each measuring 20 m by 20 m, were established between 2017 and 2018 along an elevational gradient ranging from 270 to 1470 m, with approximately 100 m intervals in elevation. For the PS method, each forest plot included in our study comprised more than fifteen standing trees, each with a diameter at breast height of at least 15 cm. From these, we randomly selected fifteen trees and harvested bryophyte samples. Previous studies has indicated that epiphytic bryophytes tend to favor the north‐facing side as well as the lower (0.3 m) and middle (1.5 m) sections of tree trunks in the subtropical forests of China (Zhao et al. 2021). Consequently, we assessed epiphytic bryophytes on each tree at heights of 0.3 and 1.5 m, oriented toward the north. For this purpose, we used a 20 × 20 cm metal frame quadrat, which was divided into 100 equal‐sized standard grids, positioned as a subplot on each sampled tree. Floristic habitat sampling (FHS) utilizes individual microhabitats within a particular type of mesohabitat as units of sampling, enabling a comprehensive inventory of plant species within a specified region. Described by Newmaster et al. (2005), this method emphasizes the survey of as many meso and microhabitats as feasible to compile an exhaustive list of species present. Expanding on this methodology, we performed FHS by methodically investigating various microhabitats such as trees, soil, logs, rocks, and stumps within a predetermined area until no additional species were identified. To achieve a representative sample of the corticolous bryophyte diversity in the plot, we sampled the bases of all trees from approximately 0.3 to 1.8 m height. Since tree canopies may harbor some bryophyte species barely present at the lower trunk (Kaufmann et al. 2019), we also collected fallen branches and trees brought down by storms. This approach allowed us to study bryophytes that might not be easily accessible from the ground, thus enhancing our understanding of their distribution and diversity within the canopy environment.

**FIGURE 1:**
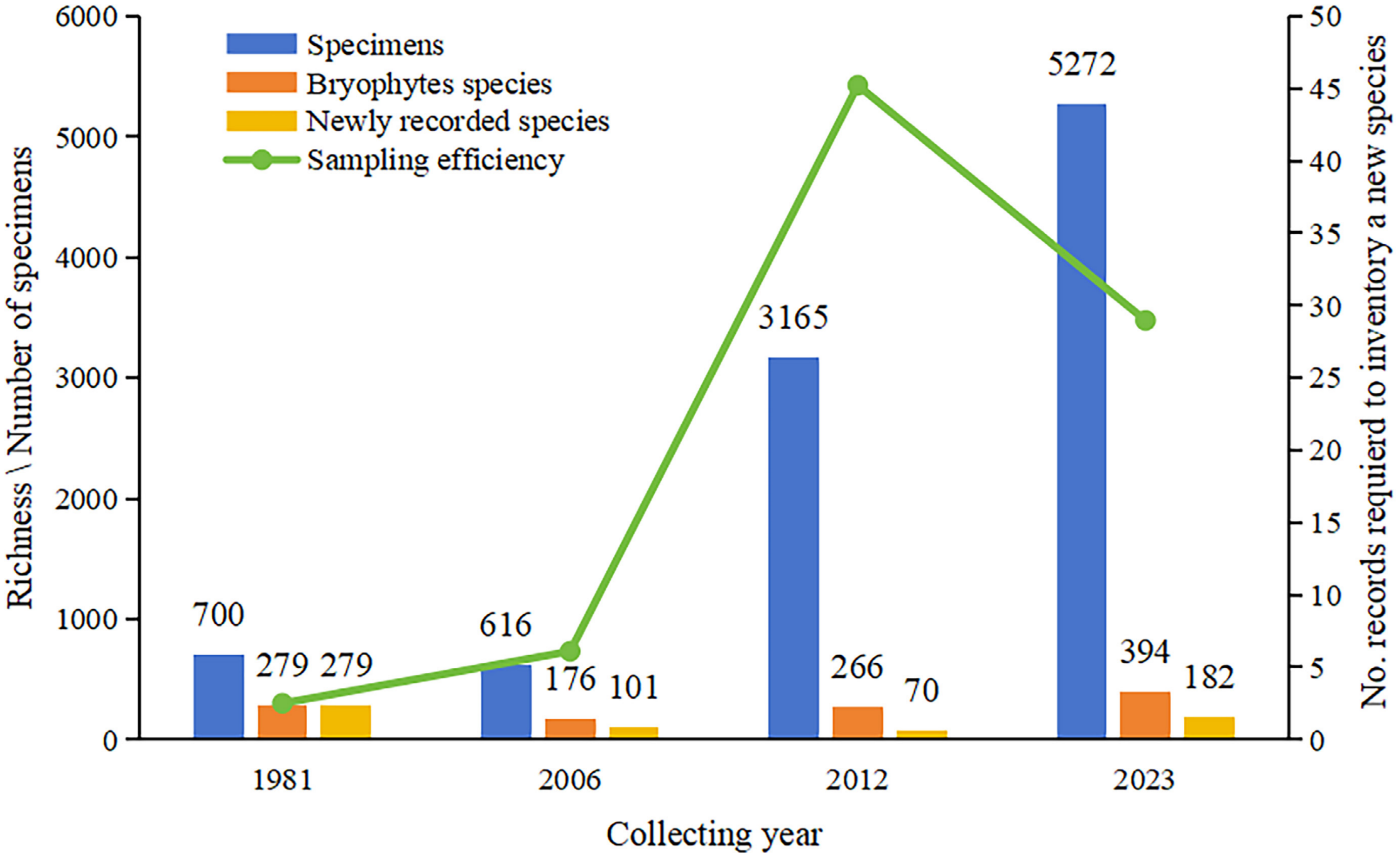
Temporal evolution in the sampling efficiency of bryophytes. The number of specimens, bryophyte species, and newly recorded species was calculated based on each independent collection.

To evaluate whether our sampling strategy is more conductive to discovering more new recorded species, we analyze the relative contribution of our sampling methods and sampling effort to the new recorded species. The species cumulative curve was used to analyze the influence of inventory effort (based on the number of specimens) on the accumulation of the 182 new recorded species. We used the freeware EstimateS 9.1.0 (Colwell 2013) to estimate the species that remain to be discovered based on species presence‐absence data of Chen et al. (2023).

All analyses were performed with R (version 4.3.0, R Core Team 2023).

## 3 Results

### 3.1 How Did the Process of Species Inventorying Change Over Time?

Survey effort in TNNR has been increasing since the first collections carried out in 1981, and the number of database records per collection was positively correlated with the number of recorded species (Figure 1). The greatest survey effort was made during the last decade. The survey effort necessary to record a newly recorded species for TNNR followed a well‐defined pattern during the period of 1981 to 2012, indicating that an increasing survey effort will be required with time. However, this temporal trend of increasing survey effort shifted after 2012, with the database entries required to document a newly recorded species dropping from 45 in 2012 to 29 in 2023 (Figure 1).

### 3.2 How Does Species Composition Change With Time?

**FIGURE 2:**
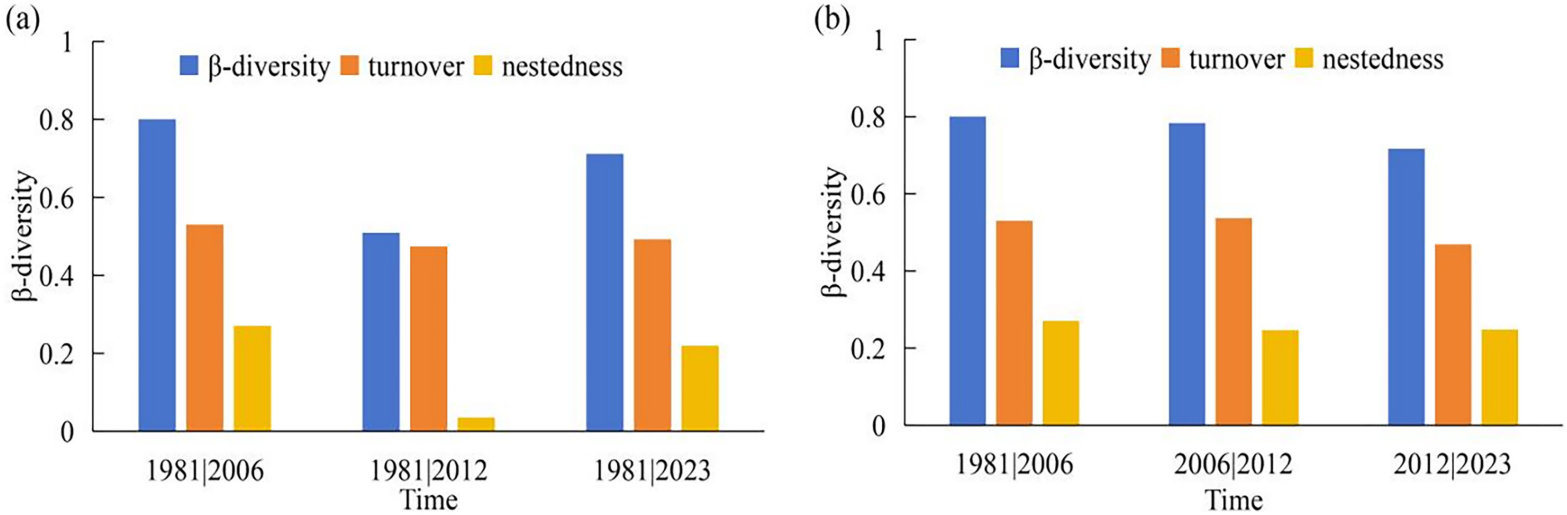
Statistical descriptions of β‐diversity components (i.e., total, turnover, and nestedness). (a) pairwise comparison of 1981 database records with subsequent inventories; (b) pairwise comparison between previous inventories.

Pairwise comparisons between the four periods (i.e., 1981 vs. 2006, 1981 vs. 2012, 1981 vs. 2023, 2006 vs. 2012, 2012 vs. 2023) all revealed a high level of total beta diversity (0.51–0.80, Figure 2). The species turnover component of total beta diversity was always higher than nestedness‐driven dissimilarity. Although the total beta diversity between 1981 and 2012 was the smallest, the species turnover component accounted for the largest portion of the overall dissimilarity, compared to the nestedness component.

### 3.3 What Are the Characteristics of New Recorded Species?

In terms of the number of newly recorded bryophytes, Lejeuneaceae (18 taxa), Neckeraceae (17 taxa), Plagiotheciaceae (12 taxa), and Mniaceae (11 taxa) are the most representative of TNNR (Table S1). The body size of these dominant families varies significantly, from small to medium in Lejeuneaceae (0.2–3 mm wide with leaves) to large in Neckeraceae (2–5 mm wide with leaves). In total, species with a narrow and wide elevational range accounted for 69.8% and 30.2% of the total number of new recorded species, respectively. Compared to non‐epiphytic bryophytes, epiphytic species are dominant among the newly recorded species, within both the narrow and wide elevational range categories (Table 1).

**TABLE 1:** The species attributes of newly recorded bryophytes.

	Range Size					
	Narrow			Wide		
	Epiphytic species	Terrestrial species	Both habitats	Epiphytic species	Terrestrial species	Both habitats
Liverworts	12 (6.6%)	6 (3.3%)	16 (8.8%)	6 (3.3%)	0 (0%)	8 (4.4%)
Mosses	40 (22.0%)	25 (13.7%)	28 (15.4%)	8 (4.4%)	3 (1.6%)	30 (16.5%)
Total	52 (28.6%)	31 (17.0%)	44 (24.2%)	14 (7.7%)	3 (1.6%)	38 (20.9%)

### 3.4 Is Our Sampling Strategy More Conductive to Discover More New Recorded Species?

**FIGURE 3:**
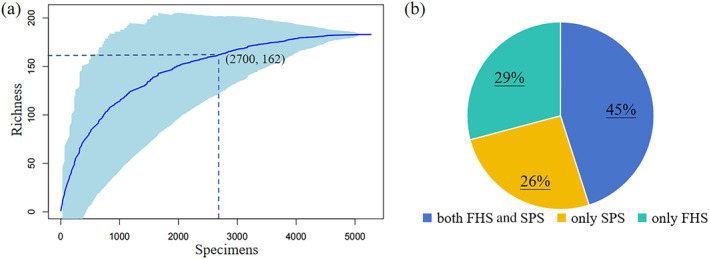
Contribution of two sampling methods and inventory intensity to newly recorded species. (a) inventory intensity based on the number of specimens. The coordinates show the number of species when the number of specimens reaches 2700; (b) contribution of two sampling methods.

The number of newly recorded species increases rapidly as the number of collected specimens increases (Figure 3a). When the number of collected specimens reached about 2700, the number of newly discovered species could reach up to 90% of the total number of species. The FHS method contributed slightly more newly recorded species than the PS method (74% vs. 71% of the total number of newly recorded species, Figure 3b). If only one of the methods (FHS method or PS method) was adopted, 26%–29% of the newly discovered species will be missed.

### 3.5 How Many Bryophytes Remain Undiscovered in TNNR, and Where Should We Look for Them?

**FIGURE 4:**
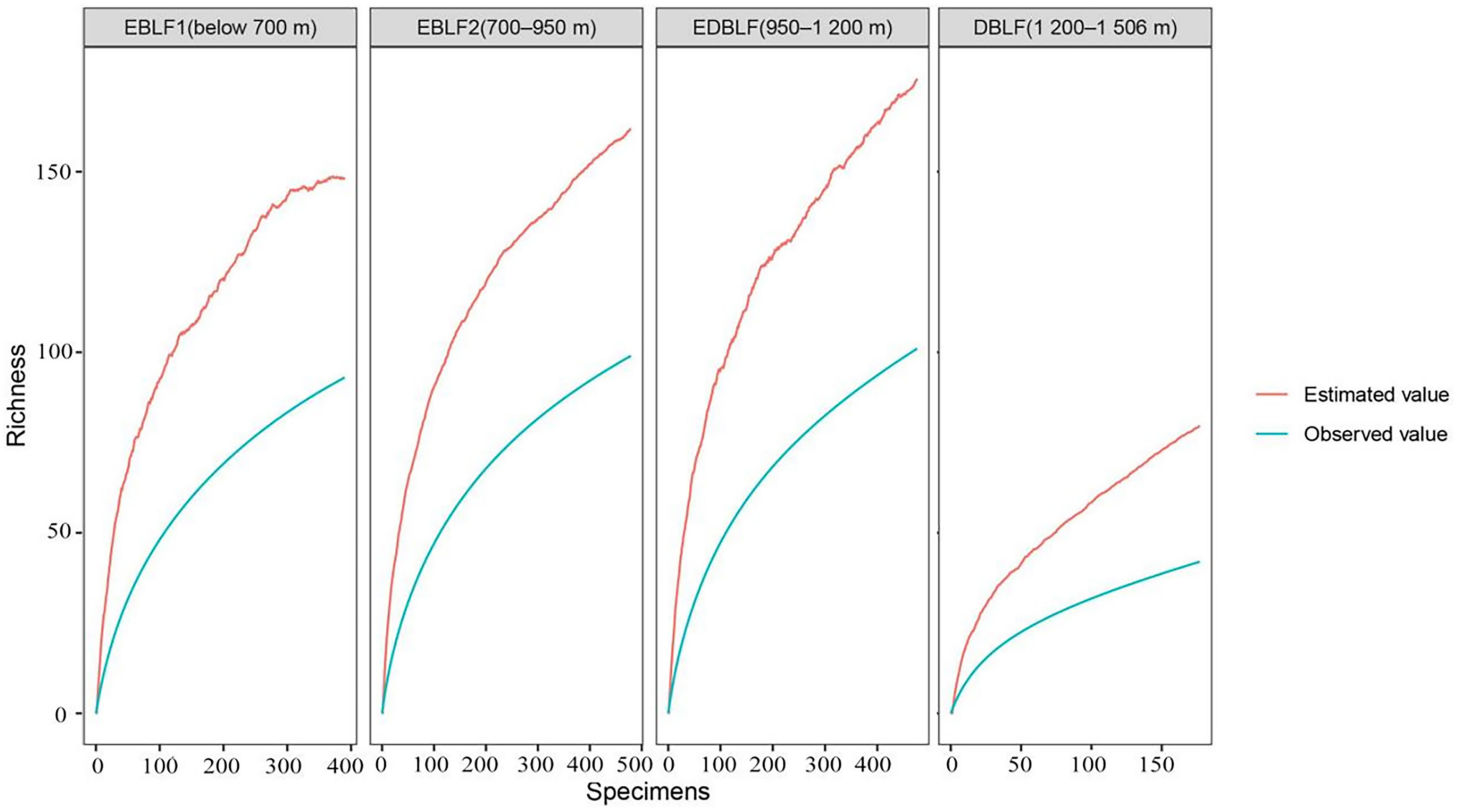
Prediction of newly recorded species in different vegetation types. EBLF1: Evergreen broad‐leaved forest zone (below 700 m); EBLF2: Evergreen broad‐leaved forest zone (700–950 m); EDBLF: Evergreen and deciduous broad‐leaved mix forest zone (950–1200 m); DBLF: Deciduous broad‐leaved forest zone (1200–1506 m).

In TNNR, we inferred that there are likely 185 bryophyte species yet to be discovered, given the present 394 species already encountered (*n*_est_ = 579). Our estimates at different vegetation types (Figure 4) showed that roughly 75 undiscovered species occur in EDBLF (*n*_obs_ = 101, *n*_est_ = 176), followed by EBLF2 (63 spp., *n*_obs_ = 99, *n*_est_ = 162), EBLF1 (55 spp., *n*_obs_ = 93, *n*_est_ = 148), and DBLF (38 spp., *n*_obs_ = 42, *n*_est_ = 80).

## 4 Discussion

Knowing the total number of species in a particular region has long been a matter of great interest, motivated by the need to provide a reference point for current and future losses of biodiversity (Mora et al. 2011). Unfortunately, obtaining an accurate number is hampered because the majority of species have yet to be discovered, primarily due to the incomplete detection of species during the field work. Certainly, this situation can also emerge due to changes in species composition, which are induced by simultaneous alterations in the environment (Dornelas et al. 2023). This study evaluates the completeness of species inventories by thoroughly compiling all accessible taxonomic and distributional information on bryophytes within the TNNR. Our findings clearly indicate that the sampling strategies employed thus far have not been sufficiently effective in capturing the existing biodiversity. This ineffectiveness is likely attributable to variations in sampling methods or the levels of sampling effort applied.

Probability of species detection can be increased by increasing sampling effort, such as sample time, sample size, and the number and experience of recorders (Archaux 2009; Archaux et al. 2006; Nilsson and Nilsson 1985). It is evident that greater survey effort correlates with a higher number of species detected in our study. In comparison to various historical collections in TNNR, the greatest survey effort was made by Chen et al. (2023). Their intensive recording effort (5272 database records) facilitated the discovery of 182 newly recorded species in the reserve. Nonetheless, even with a markedly reduced sampling effort (2700 database records), it is still feasible to discover approximately 90% of the species total (164 spp., Figure 3).

The sampling methods we employed may facilitate the discovery of more newly recorded species. In previous investigations conducted in TNNR, researchers relied on a single sampling method, either the FHS method or the PS method (Hu and Wang 1981; Li et al. 2006; Wang et al. 2012, 2013). However, it is well‐documented that every sampling method presents distinct advantages and disadvantages (Tilman 1996). The PS method proves highly effective in identifying the most common species and assessing their frequency and distribution, yet it often fails to record rare species (Vanderpoorten, Papp, and Gradstein 2010). On the other hand, the FHS method, which takes the entire mesohabitat as its basic sampling unit, offers flexibility to account for the diversity of microhabitats within a mesohabitat, thereby significantly increasing the likelihood of detecting rare species (Newmaster et al. 2005). To achieve a more comprehensive understanding of bryophyte diversity in TNNR, both PS and FHS methods were employed in the study conducted by Chen et al. (2023). Although the FHS method captured a higher mean species richness per plot compared to the PS method, the PS method demonstrated its effectiveness in the detection of smaller bryophyte species, particularly taxa belonging to the Lejeuneaceae family. Furthermore, the study revealed that relying exclusively on either the FHS or PS method would have resulted in missing 26%–29% of newly discovered species. Our finding underscores the importance of utilizing a combination of sampling methods to ensure a more complete inventory of species, capitalizing on the strengths of both approaches.

Despite the significant contribution of sampling effort and method to the discovery of newly recorded species, our data also indicated that the composition of bryophyte assemblages in TNNR has undergone changes over time. These changes may be attributed to the impacts of climate change or human modifications. Pairwise comparisons between different time periods consistently revealed a high level of species turnover, which constitutes a substantial component of total beta diversity, ranging from 65.3% to 92.0%. This indicates that the community composition of bryophytes has experienced considerable shifts over the past four decades in TNNR. It is not surprising that microscopic organisms, such as bryophytes, exhibit a rapid rate of species turnover over time, due to the fact that small organisms tend to have faster long‐term temporal turnover compared to larger organisms (Korhonen, Soininen, and Hillebrand 2010).

At local scales, species richness tends to remain remarkably constant, even though species composition can be highly variable and undergo substantial changes in response to environmental fluctuations. This stability in species richness requires the maintenance of relatively constant levels of productivity and resource availability, as well as an open system that allows for compensatory colonizations and extinctions (Brown et al. 2001). In a situation where the availability of source resources remains unchanged, the metrics that measure the total resource use by the community should also stay stable. Moreover, if changes in species composition are driven by environmental changes, these changes should exhibit a higher level of determinism than what would be expected by chance alone. In contrast, if species turnover were purely stochastic, colonizations and extinctions would be predominantly confined to rare species (Brown et al. 2001). With a current inventory of 394 species, Chen et al. (2023) have documented a significantly higher number of species compared to the average richness of historical collections (240 species). Our findings also indicate that 30.2% of the newly recorded species have a broad elevational range. This suggests that alterations in species composition in TNNR may be attributed, in part, to environmental shifts. In TNNR, there has been a 1.5°C increase in mean annual temperatures (NOAA's National Climatic Data Center, derived from 23 selected grid data). Associated with these changes have been increases and colonizations of species characteristic of epiphytic habitats. In addition, five epiphyllous liverworts, a special group of epiphytic bryophytes that are particularly sensitive to environmental change, have been recorded newly from TNNR (Tang et al. 2018). This further supports the notion that environmental conditions in TNNR have shifted, as epiphytic bryophytes display a higher ability to adapt to areas with suitable climates compared to non‐epiphytic species (Dai, Zhang, and Wang 2023).

Using the data of Chen et al. (2023), we estimated that there are about 185 species that remain undiscovered, meaning that a greater survey effort is going to be required to add newly recorded species to the inventory of TNNR. In order to complete the bryophytes inventory in TNNR, more survey efforts should be paid in the mid‐elevation of the reserve (950–1200 m), an ecotone zone composed by evergreen and deciduous broad‐leaved mixed forest, that is also predicted to harbor relatively high species richness due to overlapping range limits or due to source‐sink dynamics (Terborgh 1985; Lomolino 2001).

In summary, our results demonstrated that the species composition of bryophytes in TNNR has undergone significant shifts during the period of 1981 to 2023. It is also clear that the higher the survey effort, the greater the number of newly recorded species. When the same effort was made, an appropriate sampling methodology is crucial to accelerating newly recorded species discovery. Moreover, our study also highlights the importance of considering both the imperfect detection of species and species temporal turnover when assessing the completeness of species inventories at the local scale.

## Author Contributions

**Xue Yao:** data curation (supporting), methodology (lead), writing – original draft (lead), writing – review and editing (supporting). **Zun Dai:** data curation (lead), investigation (equal), methodology (supporting). **Yiran Wang:** data curation (supporting), writing – review and editing (supporting). **Jian Zhang:** conceptualization (lead), funding acquisition (lead), methodology (supporting), supervision (lead), writing – original draft (supporting), writing – review and editing (supporting). **Jian Wang:** conceptualization (lead), data curation (supporting), funding acquisition (supporting), investigation (lead), methodology (supporting), supervision (lead), writing – original draft (lead), writing – review and editing (supporting).

## Conflicts of Interest

The authors declare no conflicts of interest.

## Supporting information


**Table S1.** The dominant families and genera of newly added bryophytes.

## Data Availability

The datasets for this study are accessible in Supporting Information.
