# Supplementary material for: What Determines the Probability of Discovering a Species? A Study of the Completeness of Bryophyte Inventories in Tianmushan National Nature Reserve (Zhejiang, China)
Source: Ecol Evol. 2024 Nov 21;14(11):e70593. doi: 10.1002/ece3.70593 (PMC11581785; doi:10.1002/ece3.70593)
Supplement: Supplementary file 1 — Table S1. The dominant families and genera of newly added bryophytes. [file ECE3-14-e70593-s001.docx]

TABLE S1 The dominant families and attributes of newly recorded bryophytes.

| No. | Dominant families (No. species ≥ 7) | | Species size | Main habitat |
| --- | --- | --- | --- | --- |
|  | Family | No. of species |  |  |
| 1 | Lejeuneaceae | 18 (9.89%) | small to large | epiphytes |
| 2 | Neckeraceae | 17 (9.34%) | robust | tree trunks / rocks |
| 3 | Plagiotheciaceae | 12 (6.59%) | slender / robust | tree trunks / rocks / rotten logs |
| 4 | Mniaceae | 11 (6.04%) | slender and medium-size | soil |
| 5 | Frullaniaceae | 7 (3.85%) | small to medium-size | epiphytes |
| 6 | Porellaceae | 7 (3.85%) | large | epiphytes |
| 7 | Pylaisiaceae | 7 (3.85%) | slender and medium-size | epiphytes |

TABLE S2 The inventory records of each year.

| Year Species | 1981 | 2006 | 2012 | 2023 |
| --- | --- | --- | --- | --- |
| *Acrolejeunea sandvicensis* | 1 | 0 | 1 | 1 |
| *Acroporium lamprophyllum* | 0 | 1 | 0 | 0 |
| *Aerobryidium filamentosum* | 0 | 0 | 1 | 0 |
| *Amblystegium serpens* | 0 | 0 | 0 | 1 |
| *Amblystegium varium* | 0 | 0 | 1 | 0 |
| *Aneura pinguis* | 1 | 0 | 1 | 1 |
| *Anoectangium aestivum* | 0 | 0 | 1 | 0 |
| *Anoectangium stracheyanum* | 1 | 0 | 1 | 1 |
| *Anoectangium thomsonii* | 1 | 0 | 0 | 0 |
| *Anomodon giraldii* | 1 | 1 | 1 | 1 |
| *Anomodon minor* | 1 | 0 | 1 | 1 |
| *Anomodon rugelii* | 1 | 1 | 1 | 1 |
| *Anomodon viticulosus* | 1 | 0 | 0 | 0 |
| *Ardeuma recurvirostrum* | 1 | 0 | 0 | 0 |
| *Atrichum crispulum* | 1 | 0 | 1 | 1 |
| *Atrichum rhystophyllum* | 1 | 0 | 1 | 0 |
| *Atrichum undulatum* var. *gracilisetum* | 1 | 0 | 1 | 1 |
| *Atrichum yakushimense* | 0 | 0 | 1 | 0 |
| *Barbella compressiramea* | 0 | 0 | 0 | 1 |
| *Barbula gracilenta* | 0 | 0 | 1 | 0 |
| *Barbula sordida* | 0 | 0 | 1 | 0 |
| *Barbula unguiculata* | 1 | 0 | 1 | 0 |
| *Bartramia halleriana* | 0 | 1 | 0 | 0 |
| *Bartramia pomiformis* | 1 | 0 | 1 | 0 |
| *Bazzania albifolia* | 0 | 1 | 0 | 0 |
| *Bazzania bidentula* | 0 | 0 | 0 | 1 |
| *Bazzania japonica* | 1 | 0 | 1 | 1 |
| *Bazzania tridens* | 1 | 0 | 0 | 1 |
| *Blepharostoma trichophyllum* | 1 | 0 | 0 | 0 |
| *Brachymenium capitulatum* | 0 | 0 | 1 | 0 |
| *Brachymenium exile* | 0 | 0 | 1 | 0 |
| *Brachymenium nepalense* | 1 | 0 | 1 | 1 |
| *Brachytheciastrum velutinum* | 0 | 0 | 0 | 1 |
| *Brachythecium albicans* | 1 | 0 | 1 | 0 |
| *Brachythecium buchananii* | 1 | 0 | 1 | 1 |
| *Brachythecium campylothallum* | 0 | 0 | 1 | 1 |
| *Brachythecium cirrosum* | 0 | 0 | 0 | 1 |
| *Brachythecium coreanum* | 0 | 1 | 0 | 0 |
| *Brachythecium dicranoides* | 1 | 1 | 0 | 0 |
| *Brachythecium garovaglioides* | 1 | 0 | 0 | 1 |
| *Brachythecium glaciale* | 0 | 1 | 0 | 0 |
| *Brachythecium glareosum* | 1 | 0 | 1 | 0 |
| *Brachythecium moriense* | 0 | 1 | 1 | 0 |
| *Brachythecium noguchii* | 1 | 0 | 1 | 1 |
| *Brachythecium novae-angliae* | 1 | 0 | 1 | 0 |
| *Brachythecium oedipodium* | 0 | 1 | 0 | 0 |
| *Brachythecium pendulum* | 0 | 0 | 0 | 1 |
| *Brachythecium perminusculum* | 0 | 1 | 0 | 0 |
| *Brachythecium perscabrum* | 0 | 0 | 1 | 0 |
| *Brachythecium piligerum* | 1 | 1 | 1 | 1 |
| *Brachythecium procumbens* | 1 | 0 | 1 | 0 |
| *Brachythecium rivulare* | 1 | 1 | 0 | 1 |
| *Brachythecium rotaeanum* | 0 | 0 | 0 | 1 |
| *Brachythecium rutabulum* | 0 | 0 | 1 | 1 |
| *Brachythecium salebrosum* | 0 | 0 | 1 | 0 |
| *Brachythecium starkei* | 0 | 1 | 0 | 0 |
| *Brachythecium trichomitrium* | 1 | 0 | 1 | 0 |
| *Brachythecium viridefactum* | 0 | 1 | 0 | 0 |
| *Breidleria erectiuscula* | 0 | 0 | 1 | 0 |
| *Brothera leana* | 0 | 0 | 1 | 0 |
| *Brotherella curvirostris* | 0 | 0 | 0 | 1 |
| *Brotherella erythrocaulis* | 0 | 1 | 0 | 1 |
| *Brotherella falcata* | 1 | 0 | 0 | 0 |
| *Brotherella fauriei* | 1 | 1 | 1 | 1 |
| *Brotherella henonii* | 1 | 1 | 1 | 0 |
| *Brotherella henonii* var. *falcatula* | 0 | 1 | 0 | 0 |
| *Brotherella nictans* | 0 | 0 | 0 | 1 |
| *Bryhnia brachycladula* | 0 | 1 | 1 | 0 |
| *Bryhnia trichomitria* | 0 | 1 | 0 | 0 |
| *Bryum argenteum* | 1 | 0 | 0 | 0 |
| *Bryum billarderi* | 1 | 0 | 1 | 1 |
| *Bryum capillare* | 1 | 0 | 1 | 0 |
| *Bryum cyclophyllum* | 0 | 0 | 1 | 0 |
| *Calypogeia arguta* | 0 | 0 | 1 | 0 |
| *Calypogeia fissa* | 0 | 0 | 1 | 1 |
| *Calypogeia muelleriana* | 0 | 0 | 1 | 0 |
| *Calypogeia tosana* | 0 | 0 | 0 | 1 |
| *Campyliadelphus elodes* | 0 | 1 | 0 | 0 |
| *Campylium hispidulum* | 0 | 0 | 1 | 0 |
| *Campylopodium medium* | 0 | 0 | 0 | 1 |
| *Campylopus atrovirens* | 1 | 0 | 1 | 0 |
| *Campylopus fragilis* | 0 | 0 | 1 | 0 |
| *Campylopus gracilis* | 1 | 0 | 0 | 0 |
| *Campylopus schmidii* | 0 | 0 | 0 | 1 |
| *Campylopus sinensis* | 1 | 0 | 1 | 0 |
| *Cephalozia lacinulata* | 0 | 0 | 0 | 1 |
| *Cephaloziella microphylla* | 1 | 0 | 0 | 1 |
| *Cephaloziella spinicaulis* | 0 | 0 | 0 | 1 |
| *Cheilolejeunea trapezia* | 0 | 0 | 1 | 1 |
| *Cheilolejeunea xanthocarpa* | 0 | 0 | 0 | 1 |
| *Chiloscyphus polyanthos* | 1 | 0 | 1 | 1 |
| *Chrysocladium retrorsum* | 1 | 1 | 1 | 1 |
| *Cirriphyllum piliferum* | 1 | 0 | 0 | 0 |
| *Claopodium aciculum* | 1 | 1 | 1 | 1 |
| *Claopodium assurgens* | 0 | 0 | 0 | 1 |
| *Claopodium gracillimum* | 0 | 1 | 1 | 1 |
| *Claopodium pellucinerve* | 1 | 1 | 0 | 0 |
| *Clastobryum glabrescens* | 0 | 0 | 0 | 1 |
| *Climacium dendroides* | 0 | 1 | 0 | 0 |
| *Climacium japonicum* | 1 | 1 | 1 | 1 |
| *Cololejeunea japonica* | 0 | 0 | 0 | 1 |
| *Cololejeunea lanciloba* | 0 | 0 | 1 | 0 |
| *Cololejeunea longifolia* | 0 | 0 | 1 | 1 |
| *Cololejeunea pseudofloccosa* | 0 | 0 | 0 | 1 |
| *Cololejeunea raduliloba* | 0 | 0 | 0 | 1 |
| *Cololejeunea spinosa* | 0 | 0 | 0 | 1 |
| *Cololejeunea tianmuensis* | 0 | 0 | 0 | 1 |
| *Conocephalum conicum* | 1 | 0 | 1 | 1 |
| *Conocephalum japonicum* | 1 | 0 | 1 | 0 |
| *Cratoneuron filicinum* | 0 | 0 | 1 | 0 |
| *Cryphaea obovatocarpa* | 0 | 0 | 0 | 1 |
| *Ctenidium andoi* | 0 | 0 | 1 | 1 |
| *Ctenidium capillifolium* | 0 | 1 | 0 | 1 |
| *Ctenidium hastile* | 0 | 0 | 0 | 1 |
| *Ctenidium malacobolum* | 0 | 0 | 0 | 1 |
| *Ctenidium pinnatum* | 1 | 0 | 1 | 1 |
| *Ctenidium serratifolium* | 0 | 0 | 0 | 1 |
| *Cyathophorum hookerianum* | 1 | 0 | 0 | 0 |
| *Cyrto-hypnum tamariscellum* | 0 | 1 | 1 | 0 |
| *Dicranella coarctata* | 1 | 0 | 0 | 1 |
| *Dicranella heteromalla* | 1 | 0 | 1 | 1 |
| *Dicranella micro-divariata* | 0 | 0 | 0 | 1 |
| *Dicranella subulata* | 0 | 0 | 0 | 1 |
| *Dicranella varia* | 0 | 0 | 1 | 0 |
| *Dicranodontium denudatum* | 1 | 0 | 1 | 0 |
| *Dicranodontium filifolium* | 0 | 0 | 0 | 1 |
| *Dicranodontium uncinatum* | 1 | 0 | 1 | 0 |
| *Dicranoloma cylindrothecium* | 0 | 1 | 0 | 0 |
| *Dicranoloma dicarpum* | 1 | 0 | 0 | 0 |
| *Dicranoweisia crispula* | 1 | 0 | 1 | 0 |
| *Dicranum assamicum* | 0 | 0 | 0 | 1 |
| *Dicranum drummondii* | 0 | 1 | 1 | 0 |
| *Dicranum japonicum* | 1 | 1 | 1 | 0 |
| *Dicranum linzianum* | 0 | 1 | 0 | 0 |
| *Dicranum majus* | 1 | 0 | 1 | 0 |
| *Dicranum muehlenbeckii* | 1 | 0 | 1 | 1 |
| *Dicranum nipponense* | 1 | 0 | 0 | 0 |
| *Dicranum scoparium* | 1 | 1 | 1 | 1 |
| *Dicranum subporodictyon* | 0 | 0 | 0 | 1 |
| *Didymodon constrictus* | 0 | 0 | 1 | 0 |
| *Didymodon ferrugineus* | 1 | 0 | 0 | 0 |
| *Didymodon rufidulus* | 1 | 0 | 0 | 0 |
| *Diphyscium fulvifolium* | 1 | 0 | 1 | 0 |
| *Ditrichum brevidens* | 0 | 0 | 0 | 1 |
| *Ditrichum pallidum* | 1 | 0 | 1 | 1 |
| *Dolichomitra cymbifolia* | 1 | 1 | 0 | 0 |
| *Dolichomitriopsis diversiformis* | 1 | 1 | 1 | 1 |
| *Dozya japonica* | 0 | 0 | 0 | 1 |
| *Drepanocladus aduncus* var. *kneiffii* | 0 | 1 | 1 | 0 |
| *Drepanolejeunea erecta* | 0 | 0 | 0 | 1 |
| *Dumortiera hirsuta* | 1 | 0 | 1 | 0 |
| *Duthiella flaccida* | 1 | 1 | 1 | 0 |
| *Duthiella speciosissima* | 1 | 0 | 0 | 0 |
| *Ectropothecium ohosimense* | 1 | 0 | 0 | 0 |
| *Ectropothecium zollingeri* | 0 | 0 | 0 | 1 |
| *Entodon challengeri* | 0 | 0 | 1 | 0 |
| *Entodon cladorrhizans* | 1 | 1 | 1 | 1 |
| *Entodon conchophyllus* | 0 | 1 | 1 | 1 |
| *Entodon concinnus* | 1 | 0 | 1 | 0 |
| *Entodon dolichocucullatus* | 0 | 0 | 0 | 1 |
| *Entodon flavescens* | 1 | 1 | 1 | 1 |
| *Entodon giraldii* | 0 | 1 | 0 | 0 |
| *Entodon kungshanensis* | 0 | 1 | 0 | 0 |
| *Entodon longifolius* | 0 | 1 | 1 | 1 |
| *Entodon luridus* | 1 | 0 | 0 | 1 |
| *Entodon macropodus* | 1 | 1 | 1 | 1 |
| *Entodon obtusatus* | 0 | 1 | 0 | 1 |
| *Entodon plicatus* | 0 | 0 | 0 | 1 |
| *Entodon prorepens* | 0 | 1 | 1 | 1 |
| *Entodon pulchellus* | 0 | 0 | 1 | 1 |
| *Entodon scariosus* | 0 | 0 | 0 | 1 |
| *Entodon schleicheri* | 0 | 1 | 1 | 0 |
| *Entodon smaragdinus* | 0 | 1 | 0 | 1 |
| *Entodon sullivantii* | 1 | 1 | 1 | 1 |
| *Entodon taiwanensis* | 0 | 0 | 0 | 1 |
| *Entodon viridulus* | 0 | 1 | 0 | 1 |
| *Entodon yunnanensis* | 0 | 0 | 0 | 1 |
| *Eurhynchiadelphus eustegia* | 1 | 0 | 0 | 1 |
| *Eurhynchium angustirete* | 1 | 0 | 1 | 0 |
| *Eurhynchium kirishimense* | 1 | 1 | 0 | 1 |
| *Fabronia ciliaris* | 0 | 1 | 0 | 0 |
| *Fabronia matsumurae* | 1 | 0 | 0 | 0 |
| *Fauriella tenerrima* | 1 | 1 | 1 | 1 |
| *Fauriella tenuis* | 0 | 0 | 0 | 1 |
| *Fissidens anomalus* | 0 | 1 | 0 | 1 |
| *Fissidens bryoides* | 1 | 0 | 1 | 0 |
| *Fissidens crassipes* | 0 | 1 | 0 | 0 |
| *Fissidens crispulus* | 0 | 1 | 1 | 1 |
| *Fissidens dubius* | 1 | 1 | 1 | 1 |
| *Fissidens gardneri* | 0 | 0 | 1 | 0 |
| *Fissidens geminiflorus* | 0 | 1 | 0 | 0 |
| *Fissidens grandifrons* | 0 | 1 | 0 | 0 |
| *Fissidens guangdongensis* | 0 | 0 | 0 | 1 |
| *Fissidens gymnogynus* | 0 | 1 | 1 | 1 |
| *Fissidens involutus* | 0 | 1 | 0 | 0 |
| *Fissidens javanicus* | 0 | 0 | 0 | 1 |
| *Fissidens nobilis* | 1 | 0 | 1 | 0 |
| *Fissidens oblongifolius* | 0 | 0 | 0 | 1 |
| *Fissidens obscurus* | 0 | 1 | 0 | 1 |
| *Fissidens osmundoides* | 1 | 0 | 1 | 0 |
| *Fissidens pellucidus* | 0 | 0 | 0 | 1 |
| *Fissidens perdecurrens* | 0 | 1 | 0 | 0 |
| *Fissidens polypodioides* | 0 | 1 | 0 | 1 |
| *Fissidens taxifolius* | 1 | 0 | 1 | 1 |
| *Fissidens teysmannianus* | 0 | 1 | 1 | 1 |
| *Floribundaria floribunda* | 1 | 0 | 0 | 1 |
| *Floribundaria pseudofloribunda* | 0 | 0 | 0 | 1 |
| *Forsstroemia goughiana* | 0 | 0 | 0 | 1 |
| *Forsstroemia producta* | 0 | 0 | 1 | 0 |
| *Forsstroemia trichomitria* | 0 | 1 | 0 | 1 |
| *Forsstroemia yezoana* | 1 | 1 | 1 | 1 |
| *Frullania amplicrania* | 0 | 0 | 0 | 1 |
| *Frullania aoshimensis* | 0 | 0 | 0 | 1 |
| *Frullania bolanderi* | 0 | 1 | 1 | 1 |
| *Frullania davurica* | 1 | 0 | 0 | 1 |
| *Frullania fuscovirens* | 0 | 0 | 1 | 1 |
| *Frullania hamatiloba* | 0 | 0 | 0 | 1 |
| *Frullania inouei* | 0 | 1 | 0 | 1 |
| *Frullania moniliata* | 1 | 1 | 1 | 1 |
| *Frullania monocera* var. *acutiloba* | 0 | 0 | 0 | 1 |
| *Frullania muscicola* | 1 | 0 | 1 | 1 |
| *Frullania parvistipula* | 1 | 0 | 1 | 1 |
| *Frullania polyptera* | 0 | 1 | 0 | 0 |
| *Frullania schensiana* | 0 | 0 | 0 | 1 |
| *Frullania sinensis* | 1 | 0 | 0 | 1 |
| *Frullania tamarisci* | 1 | 0 | 0 | 1 |
| *Frullania taradakensis* | 0 | 0 | 0 | 1 |
| *Frullania valida* | 1 | 0 | 0 | 0 |
| *Funaria attenuata* | 1 | 0 | 0 | 0 |
| *Funaria hygrometrica* | 1 | 0 | 1 | 0 |
| *Glyphomitrium calycinum* | 0 | 0 | 0 | 1 |
| *Glyphomitrium humillimum* | 0 | 0 | 1 | 0 |
| *Glyphomitrium minutissimum* | 0 | 0 | 0 | 1 |
| *Gollania clarescens* | 0 | 0 | 0 | 1 |
| *Gollania robusta* | 0 | 0 | 0 | 1 |
| *Gollania ruginosa* | 1 | 1 | 1 | 1 |
| *Gollania varians* | 1 | 0 | 0 | 0 |
| *Grimmia atrata* | 0 | 1 | 0 | 0 |
| *Grimmia donniana* | 1 | 0 | 0 | 0 |
| *Grimmia elatior* | 1 | 0 | 0 | 0 |
| *Grimmia handelii* | 0 | 1 | 0 | 0 |
| *Grimmia incurva* | 0 | 1 | 0 | 1 |
| *Grimmia longirostris* | 1 | 1 | 1 | 0 |
| *Grimmia macrotheca* | 0 | 1 | 0 | 0 |
| *Grimmia ovalis* | 1 | 0 | 0 | 0 |
| *Grimmia pilifera* | 1 | 0 | 1 | 1 |
| *Gymnostomum aeruginosum* | 1 | 0 | 0 | 0 |
| *Gymnostomum calcareum* | 0 | 0 | 1 | 0 |
| *Haplocladium angustifolium* | 1 | 1 | 1 | 1 |
| *Haplocladium microphyllum* | 1 | 0 | 1 | 0 |
| *Haplocladium strictulum* | 1 | 1 | 0 | 0 |
| *Haplohymenium flagelliforme* | 0 | 0 | 0 | 1 |
| *Haplohymenium pseudo-triste* | 0 | 1 | 0 | 1 |
| *Haplohymenium sieboldii* | 0 | 0 | 0 | 1 |
| *Haplohymenium triste* | 1 | 0 | 1 | 1 |
| *Hedwigia ciliata* | 1 | 1 | 1 | 1 |
| *Herbertus dicranus* | 1 | 0 | 0 | 0 |
| *Herbertus longifissus* | 1 | 0 | 0 | 0 |
| *Herpetineuron toccoae* | 1 | 1 | 1 | 1 |
| *Herzogiella perrobusta* | 0 | 0 | 0 | 1 |
| *Herzogiella turfacea* | 0 | 0 | 0 | 1 |
| *Heteroscyphus argutus* | 0 | 1 | 0 | 1 |
| *Heteroscyphus coalitus* | 0 | 1 | 0 | 1 |
| *Heteroscyphus planus* | 1 | 0 | 1 | 1 |
| *Heteroscyphus zollingeri* | 1 | 0 | 1 | 1 |
| *Homalia trichomanoides* | 0 | 0 | 0 | 1 |
| *Homaliadelphus sharpii* var. *rotundatus* | 0 | 0 | 0 | 1 |
| *Homaliadelphus targionianus* | 1 | 1 | 1 | 1 |
| *Homaliodendron crassinervium* | 0 | 0 | 0 | 1 |
| *Homaliodendron flabellatum* | 0 | 0 | 0 | 1 |
| *Homaliodendron ligulaefolium* | 0 | 0 | 0 | 1 |
| *Homaliodendron microdendron* | 0 | 1 | 0 | 0 |
| *Homaliodendron montagneanum* | 0 | 0 | 0 | 1 |
| *Homaliodendron papillosum* | 0 | 0 | 0 | 1 |
| *Homaliodendron scalpellifolium* | 1 | 1 | 1 | 1 |
| *Homalothecium laevisetum* | 1 | 1 | 1 | 1 |
| *Homalothecium leucodonticaule* | 1 | 0 | 1 | 0 |
| *Homalothecium lutescens* | 1 | 0 | 0 | 0 |
| *Homomallium connexum* | 1 | 0 | 1 | 0 |
| *Homomallium incurvatum* | 0 | 1 | 1 | 0 |
| *Homomallium japonicoadnatum* | 1 | 0 | 1 | 0 |
| *Homomallium plagiangium* | 0 | 0 | 0 | 1 |
| *Homomallium simlaense* | 0 | 0 | 0 | 1 |
| *Hondaella caperata* | 0 | 0 | 0 | 1 |
| *Hookeria acutifolia* | 1 | 0 | 1 | 0 |
| *Hydrogonium orientale* | 0 | 0 | 1 | 0 |
| *Hygrohypnum ochraceum* | 1 | 0 | 0 | 0 |
| *Hygrohypnum smithii* | 0 | 1 | 0 | 0 |
| *Hyophila involuta* | 1 | 0 | 1 | 0 |
| *Hyophila javanica* | 0 | 0 | 1 | 0 |
| *Hyophila propagulifera* | 0 | 0 | 1 | 0 |
| *Hypnum calcicola* | 0 | 0 | 0 | 1 |
| *Hypnum callichroum* | 0 | 1 | 0 | 0 |
| *Hypnum cupressiforme* | 1 | 0 | 1 | 1 |
| *Hypnum dieckii* | 0 | 1 | 0 | 0 |
| *Hypnum fertile* | 0 | 0 | 0 | 1 |
| *Hypnum fujiyamae* | 0 | 0 | 0 | 1 |
| *Hypnum hamulosum* | 1 | 0 | 1 | 1 |
| *Hypnum leptothallum* | 1 | 0 | 1 | 1 |
| *Hypnum macrogynum* | 0 | 0 | 1 | 0 |
| *Hypnum oldhamii* | 0 | 0 | 1 | 1 |
| *Hypnum pallescens* | 0 | 0 | 0 | 1 |
| *Hypnum plumaeforme* | 1 | 0 | 1 | 1 |
| *Hypnum revolutum* | 0 | 1 | 0 | 0 |
| *Hypopterygium flavolimbatum* | 1 | 1 | 1 | 1 |
| *Isodrepanium arcuatum* | 0 | 0 | 0 | 1 |
| *Isopterygiopsis muelleriana* | 0 | 0 | 0 | 1 |
| *Isopterygiopsis pulchella* | 0 | 0 | 0 | 1 |
| *Isopterygium albescens* | 1 | 0 | 1 | 1 |
| *Isopterygium bancanum* | 1 | 0 | 0 | 1 |
| *Isopterygium minutirameum* | 0 | 0 | 0 | 1 |
| *Isothecium alopecuroides* | 0 | 1 | 0 | 0 |
| *Isothecium subdiversiforme* | 0 | 0 | 1 | 0 |
| *Jungermannia atrovirens* | 1 | 0 | 0 | 0 |
| *Jungermannia exsertifolia* | 0 | 0 | 1 | 0 |
| *Jungermannia pumila* | 1 | 0 | 1 | 1 |
| *Kiaeria glacialis* | 1 | 0 | 0 | 0 |
| *Kurzia sylvatica* | 0 | 0 | 0 | 1 |
| *Lejeunea anisophylla* | 0 | 0 | 0 | 1 |
| *Lejeunea curviloba* | 0 | 0 | 0 | 1 |
| *Lejeunea flava* | 0 | 0 | 0 | 1 |
| *Lejeunea japonica* | 1 | 0 | 1 | 1 |
| *Lejeunea obscura* | 0 | 0 | 0 | 1 |
| *Lejeunea parva* | 1 | 0 | 0 | 1 |
| *Lejeunea tuberculosa* | 0 | 0 | 0 | 1 |
| *Lepidozia reptans* | 1 | 0 | 1 | 1 |
| *Leptopterigynandrum tenellum* | 0 | 0 | 0 | 1 |
| *Lescuraea saxicola* | 0 | 0 | 0 | 1 |
| *Leskeella nervosa* | 1 | 0 | 1 | 1 |
| *Leucobryum bowringii* | 0 | 0 | 0 | 1 |
| *Leucobryum chlorophyllosum* | 1 | 1 | 1 | 1 |
| *Leucobryum glaucum* | 1 | 0 | 1 | 0 |
| *Leucobryum humillimum* | 0 | 0 | 0 | 1 |
| *Leucobryum javense* | 1 | 0 | 0 | 0 |
| *Leucobryum juniperoideum* | 1 | 1 | 1 | 0 |
| *Leucodon secundus* | 0 | 0 | 0 | 1 |
| *Leucodon sinensis* | 1 | 0 | 1 | 1 |
| *Leucodon subulatus* | 0 | 0 | 0 | 1 |
| *Lindbergia sinensis* | 1 | 1 | 1 | 1 |
| *Liochlaena lanceolata* | 1 | 0 | 1 | 0 |
| *Loeskeobryum cavifolium* | 1 | 1 | 0 | 0 |
| *Lophocolea bidentata* | 1 | 1 | 1 | 1 |
| *Lophocolea compacta* | 0 | 0 | 0 | 1 |
| *Lophocolea heterophylla* | 1 | 0 | 1 | 0 |
| *Lophocolea horikawana* | 1 | 0 | 0 | 0 |
| *Lophocolea minor* | 1 | 0 | 1 | 1 |
| *Macromitrium cavaleriei* | 0 | 1 | 0 | 0 |
| *Macromitrium ferriei* | 1 | 0 | 1 | 1 |
| *Macromitrium gymnostomum* | 1 | 0 | 0 | 1 |
| *Macromitrium japonicum* | 1 | 0 | 0 | 1 |
| *Macromitrium microstomum* | 0 | 0 | 0 | 1 |
| *Macromitrium moorcroftii* | 0 | 0 | 0 | 1 |
| *Macromitrium prolongatum* | 0 | 0 | 0 | 1 |
| *Macromitrium tosae* | 0 | 0 | 1 | 0 |
| *Marchantia paleacea* subsp. *diptera* | 1 | 0 | 0 | 0 |
| *Marchantia polymorpha* | 1 | 0 | 1 | 0 |
| *Mesonodon flavescens* | 0 | 0 | 0 | 1 |
| *Mesoptychia bantriensis* | 0 | 0 | 1 | 0 |
| *Meteorium atrovariegatum* | 1 | 0 | 1 | 0 |
| *Meteorium buchananii* | 1 | 0 | 0 | 1 |
| *Meteorium papillarioides* | 1 | 0 | 1 | 0 |
| *Meteorium polytrichum* | 0 | 1 | 0 | 1 |
| *Meteorium subpolytrichum* | 1 | 1 | 0 | 1 |
| *Metzgeria conjugata* | 1 | 1 | 0 | 0 |
| *Metzgeria consanguinea* | 0 | 1 | 0 | 1 |
| *Metzgeria crassipilis* | 0 | 1 | 0 | 0 |
| *Metzgeria furcata* | 1 | 0 | 1 | 0 |
| *Microcampylopus khasianus* | 0 | 1 | 0 | 0 |
| *Microlejeunea punctiformis* | 0 | 0 | 0 | 1 |
| *Microlejeunea ulicina* | 0 | 0 | 1 | 1 |
| *Miyabea fruticella* | 1 | 1 | 0 | 1 |
| *Mnium heterophyllum* | 1 | 0 | 1 | 0 |
| *Mnium hornum* | 0 | 0 | 0 | 1 |
| *Mnium laevinerve* | 1 | 1 | 1 | 1 |
| *Mnium lycopodioides* | 0 | 0 | 0 | 1 |
| *Mnium marginatum* | 0 | 0 | 0 | 1 |
| *Mnium spinosum* | 1 | 0 | 0 | 1 |
| *Myuroclada longiramea* | 1 | 1 | 1 | 1 |
| *Myuroclada maximowiczii* | 1 | 1 | 1 | 0 |
| *Neckera borealis* | 0 | 0 | 0 | 1 |
| *Neckera decurrens* | 0 | 0 | 0 | 1 |
| *Neckera fauriei* | 0 | 1 | 0 | 0 |
| *Neckera flexiramea* | 1 | 0 | 1 | 1 |
| *Neckera humilis* | 0 | 0 | 0 | 1 |
| *Neckera laevidens* | 0 | 0 | 0 | 1 |
| *Neckera neckeroides* | 0 | 0 | 0 | 1 |
| *Neckera pennata* | 1 | 0 | 0 | 1 |
| *Neckera perpinnata* | 0 | 0 | 0 | 1 |
| *Neckeropsis nitidula* | 1 | 0 | 1 | 1 |
| *Neodicladiella flagellifera* | 1 | 0 | 1 | 0 |
| *Neodicladiella pendula* | 0 | 0 | 1 | 1 |
| *Niphotrichum japonicum* | 1 | 0 | 1 | 0 |
| *Nowellia curvifolia* | 1 | 0 | 0 | 1 |
| *Odontoschisma denudatum* | 0 | 0 | 0 | 1 |
| *Okamuraea brachydictyon* | 0 | 1 | 0 | 1 |
| *Okamuraea brevipes* | 0 | 1 | 0 | 0 |
| *Okamuraea hakoniensis* | 0 | 1 | 0 | 1 |
| *Oncophorus crispifolius* | 0 | 0 | 1 | 0 |
| *Orthomnion javense* | 0 | 0 | 0 | 1 |
| *Orthotrichum consobrinum* | 0 | 1 | 0 | 0 |
| *Orthotrichum speciosum* | 1 | 0 | 1 | 0 |
| *Oxyrrhynchium hians* | 0 | 0 | 1 | 0 |
| *Oxyrrhynchium latifolium* | 0 | 0 | 1 | 0 |
| *Oxyrrhynchium laxirete* | 1 | 1 | 1 | 1 |
| *Oxyrrhynchium savatieri* | 0 | 1 | 1 | 0 |
| *Palamocladium euchloron* | 0 | 1 | 0 | 1 |
| *Palamocladium leskeoides* | 1 | 1 | 1 | 1 |
| *Pallavicinia lyellii* | 1 | 0 | 0 | 0 |
| *Paraleucobryum enerve* | 0 | 0 | 0 | 1 |
| *Paraleucobryum longifolium* | 1 | 0 | 1 | 0 |
| *Pelekium bonianum* | 0 | 0 | 0 | 1 |
| *Pelekium gratum* | 0 | 1 | 0 | 0 |
| *Pelekium pygmaeum* | 1 | 1 | 1 | 1 |
| *Pellia epiphylla* | 0 | 0 | 0 | 1 |
| *Pellia neesiana* | 1 | 0 | 1 | 0 |
| *Phaeoceros laevis* | 1 | 0 | 1 | 0 |
| *Philonotis fontana* | 1 | 0 | 1 | 0 |
| *Philonotis mollis* | 1 | 0 | 0 | 0 |
| *Physcomitrium courtoisii* | 1 | 0 | 1 | 0 |
| *Physcomitrium eurystomum* | 1 | 0 | 1 | 0 |
| *Pilotrichopsis dentata* | 1 | 1 | 1 | 0 |
| *Pinnatella alopecuroides* | 1 | 0 | 0 | 0 |
| *Plagiochasma intermedium* | 1 | 0 | 0 | 0 |
| *Plagiochila chinensis* | 1 | 1 | 0 | 0 |
| *Plagiochila corticola* | 0 | 1 | 0 | 0 |
| *Plagiochila furcifolia* | 1 | 0 | 1 | 1 |
| *Plagiochila gracilis* | 0 | 1 | 0 | 0 |
| *Plagiochila junghuhniana* | 0 | 0 | 0 | 1 |
| *Plagiochila nepalensis* | 0 | 0 | 0 | 1 |
| *Plagiochila ovalifolia* | 1 | 0 | 1 | 0 |
| *Plagiochila parvifolia* | 0 | 0 | 0 | 1 |
| *Plagiochila pulcherrima* | 0 | 0 | 1 | 0 |
| *Plagiochila sciophila* | 1 | 1 | 1 | 1 |
| *Plagiomnium acutum* | 1 | 1 | 1 | 1 |
| *Plagiomnium arbusculum* | 0 | 1 | 0 | 0 |
| *Plagiomnium confertidens* | 0 | 0 | 0 | 1 |
| *Plagiomnium cuspidatum* | 1 | 1 | 1 | 1 |
| *Plagiomnium ellipticum* | 0 | 0 | 0 | 1 |
| *Plagiomnium japonicum* | 0 | 1 | 0 | 0 |
| *Plagiomnium maximoviczii* | 1 | 1 | 1 | 1 |
| *Plagiomnium medium* | 0 | 1 | 0 | 1 |
| *Plagiomnium rhynchophorum* | 0 | 1 | 0 | 1 |
| *Plagiomnium rostratum* | 1 | 1 | 1 | 1 |
| *Plagiomnium succulentum* | 0 | 0 | 0 | 1 |
| *Plagiomnium venustum* | 0 | 0 | 0 | 1 |
| *Plagiomnium vesicatum* | 0 | 1 | 0 | 0 |
| *Plagiothecium cavifolium* | 0 | 0 | 0 | 1 |
| *Plagiothecium cavifolium* var. *fallax* | 0 | 0 | 0 | 1 |
| *Plagiothecium denticulatum* | 0 | 0 | 0 | 1 |
| *Plagiothecium euryphyllum* | 1 | 1 | 1 | 1 |
| *Plagiothecium formosicum* | 0 | 1 | 0 | 1 |
| *Plagiothecium neckeroideum* | 1 | 0 | 0 | 1 |
| *Plagiothecium nemorale* | 1 | 1 | 1 | 1 |
| *Plagiothecium platyphyllum* | 0 | 0 | 0 | 1 |
| *Plagiothecium succulentum* | 0 | 0 | 0 | 1 |
| *Platydictya subtilis* | 0 | 0 | 0 | 1 |
| *Platygyriella aurea* | 0 | 1 | 0 | 0 |
| *Pogonatum inflexum* | 1 | 0 | 1 | 1 |
| *Pogonatum spinulosum* | 1 | 0 | 0 | 0 |
| *Pohlia flexuosa* | 0 | 0 | 1 | 0 |
| *Polytrichastrum formosum* | 1 | 0 | 1 | 0 |
| *Polytrichum juniperinum* | 1 | 0 | 0 | 0 |
| *Porella acutifolia* | 1 | 0 | 0 | 1 |
| *Porella acutifolia* subsp. *tosana* | 0 | 0 | 0 | 1 |
| *Porella caespitans* | 1 | 0 | 0 | 1 |
| *Porella caespitans* var. *cordifolia* | 0 | 1 | 0 | 1 |
| *Porella caespitans* var. *nipponica* | 0 | 0 | 0 | 1 |
| *Porella chinensis* | 1 | 0 | 1 | 1 |
| *Porella densifolia* | 1 | 0 | 0 | 1 |
| *Porella densifolia* subsp. *appendiculata* | 1 | 0 | 0 | 1 |
| *Porella densifolia* subsp. *fallax* | 0 | 0 | 0 | 1 |
| *Porella gracillima* | 0 | 0 | 0 | 1 |
| *Porella grandiloba* | 0 | 0 | 0 | 1 |
| *Porella japonica* | 0 | 0 | 0 | 1 |
| *Porella nitens* | 0 | 1 | 0 | 1 |
| *Porella obtusata* var. *macroloba* | 1 | 1 | 1 | 0 |
| *Porella obtusiloba* | 0 | 1 | 0 | 1 |
| *Porella perrottetiana* | 1 | 1 | 0 | 0 |
| *Porella plumosa* | 1 | 0 | 0 | 0 |
| *Porella revoluta* | 0 | 0 | 1 | 1 |
| *Porella ulophylla* | 1 | 0 | 1 | 1 |
| *Porella vernicosa* | 0 | 1 | 0 | 0 |
| *Pseudobarbella levieri* | 0 | 0 | 1 | 1 |
| *Pseudoleskeella catenulata* | 1 | 0 | 1 | 1 |
| *Pseudoleskeella papillosa* | 0 | 0 | 0 | 1 |
| *Pseudoleskeella tectorum* | 1 | 0 | 0 | 1 |
| *Pseudoleskeopsis tosana* | 0 | 1 | 0 | 0 |
| *Pseudoleskeopsis zippelii* | 1 | 1 | 1 | 1 |
| *Pseudospiridentopsis horrida* | 1 | 0 | 0 | 0 |
| *Pseudosymblepharis angustata* | 1 | 0 | 1 | 1 |
| *Pseudotaxiphyllum densum* | 0 | 0 | 0 | 1 |
| *Pseudotaxiphyllum distichaceum* var. *obtusifolium* | 1 | 0 | 0 | 0 |
| *Pseudotaxiphyllum maebarae* | 0 | 0 | 1 | 0 |
| *Pseudotaxiphyllum pohliaecarpum* | 1 | 1 | 1 | 1 |
| *Pterogoniadelphus esquirolii* | 1 | 0 | 0 | 0 |
| *Ptychanthus striatus* | 1 | 1 | 0 | 1 |
| *Ptychomitrium dentatum* | 1 | 0 | 1 | 0 |
| *Ptychomitrium linearifolium* | 1 | 0 | 1 | 1 |
| *Ptychomitrium sinense* | 1 | 0 | 1 | 0 |
| *Pylaisia polyantha* | 1 | 0 | 1 | 1 |
| *Pylaisia selwynii* | 0 | 0 | 0 | 1 |
| *Pylaisiadelpha tenuirostris* | 1 | 0 | 1 | 1 |
| *Pylaisiadelpha tristoviridis* | 1 | 1 | 1 | 1 |
| *Pylaisiadelpha yokohamae* | 1 | 1 | 1 | 1 |
| *Racomitrium anomodontoides* | 1 | 0 | 1 | 0 |
| *Racomitrium nitidulum* | 0 | 0 | 0 | 1 |
| *Racopilum cuspidigerum* | 1 | 0 | 0 | 0 |
| *Radula cavifolia* | 0 | 0 | 1 | 1 |
| *Radula complanata* | 1 | 0 | 1 | 1 |
| *Radula japonica* | 0 | 1 | 0 | 1 |
| *Radula javanica* | 0 | 1 | 0 | 1 |
| *Radula kojana* | 1 | 0 | 1 | 1 |
| *Radula lindenbergiana* | 0 | 0 | 1 | 0 |
| *Radula madagascariensis* | 0 | 0 | 0 | 1 |
| *Radula tokiensis* | 0 | 0 | 0 | 1 |
| *Reboulia hemisphaerica* | 1 | 0 | 0 | 0 |
| *Rhizomnium punctatum* | 0 | 0 | 0 | 1 |
| *Rhizomnium striatulum* | 0 | 0 | 0 | 1 |
| *Rhodobryum giganteum* | 1 | 1 | 1 | 1 |
| *Rhynchostegiella japonica* | 0 | 0 | 0 | 1 |
| *Rhynchostegiella laeviseta* | 1 | 0 | 1 | 1 |
| *Rhynchostegium contractum* | 0 | 0 | 1 | 1 |
| *Rhynchostegium fauriei* | 0 | 0 | 1 | 1 |
| *Rhynchostegium inclinatum* | 1 | 0 | 0 | 1 |
| *Rhynchostegium ovalifolium* | 0 | 1 | 1 | 1 |
| *Rhynchostegium pallidifolium* | 1 | 0 | 1 | 1 |
| *Rhynchostegium riparioides* | 1 | 0 | 1 | 1 |
| *Rhynchostegium subspeciosum* | 1 | 0 | 1 | 1 |
| *Rhytidiadelphus squarrosus* | 1 | 0 | 0 | 0 |
| *Rhytidiadelphus triquetrus* | 0 | 1 | 0 | 0 |
| *Riccardia latifrons* | 0 | 0 | 0 | 1 |
| *Riccardia multifida* | 1 | 0 | 0 | 1 |
| *Riccardia palmata* | 1 | 0 | 1 | 1 |
| *Riccia fluitans* | 1 | 0 | 0 | 0 |
| *Riccia glauca* | 1 | 0 | 0 | 0 |
| *Ricciocarpos natans* | 1 | 0 | 0 | 0 |
| *Scabridens sinensis* | 0 | 0 | 0 | 1 |
| *Scapania undulata* | 0 | 0 | 1 | 0 |
| *Schistidium apocarpum* | 1 | 0 | 1 | 0 |
| *Schistidium rivulare* | 0 | 0 | 0 | 1 |
| *Schlotheimia grevilleana* | 1 | 0 | 0 | 0 |
| *Schwetschkea courtoisii* | 0 | 0 | 1 | 0 |
| *Schwetschkea laxa* | 0 | 0 | 1 | 0 |
| *Schwetschkeopsis fabronia* | 1 | 0 | 1 | 1 |
| *Sciuro-hypnum plumosum* | 1 | 1 | 1 | 1 |
| *Sciuro-hypnum populeum* | 1 | 0 | 1 | 0 |
| *Sciuro-hypnum pulchellum* | 0 | 0 | 1 | 1 |
| *Sciuro-hypnum reflexum* | 1 | 0 | 0 | 1 |
| *Sciuro-hypnum uncinifolium* | 0 | 0 | 1 | 0 |
| *Sematophyllum subhumile* | 1 | 0 | 1 | 1 |
| *Sematophyllum subpinnatum* | 0 | 1 | 0 | 1 |
| *Solenostoma handelii* | 0 | 0 | 1 | 0 |
| *Solenostoma truncatum* | 1 | 0 | 0 | 0 |
| *Solmsiella biseriata* | 0 | 0 | 0 | 1 |
| *Sphaerotheciella sphaerocarpa* | 0 | 0 | 0 | 1 |
| *Sphagnum palustre* | 1 | 0 | 0 | 0 |
| *Spruceanthus falcatus* | 0 | 0 | 0 | 1 |
| *Spruceanthus kiushianus* | 0 | 0 | 0 | 1 |
| *Spruceanthus planifolius* | 0 | 0 | 0 | 1 |
| *Spruceanthus semirepandus* | 0 | 0 | 0 | 1 |
| *Symphyodon perrottetii* | 0 | 1 | 0 | 0 |
| *Syzygiella autumnalis* | 0 | 0 | 0 | 1 |
| *Taiwanobryum crenulatum* | 0 | 0 | 0 | 1 |
| *Taiwanobryum speciosum* | 1 | 0 | 0 | 1 |
| *Taxiphyllum alternans* | 0 | 0 | 0 | 1 |
| *Taxiphyllum aomoriense* | 1 | 0 | 1 | 1 |
| *Taxiphyllum cuspidifolium* | 0 | 0 | 0 | 1 |
| *Taxiphyllum giraldii* | 0 | 1 | 0 | 0 |
| *Taxiphyllum taxirameum* | 1 | 1 | 1 | 1 |
| *Thamnobryum subseriatum* | 1 | 1 | 1 | 1 |
| *Thamnobryum subserratum* | 0 | 0 | 0 | 1 |
| *Thuidium assimile* | 0 | 1 | 0 | 1 |
| *Thuidium cymbifolium* | 1 | 1 | 1 | 1 |
| *Thuidium delicatulum* | 0 | 1 | 0 | 1 |
| *Thuidium glaucinoides* | 0 | 0 | 0 | 1 |
| *Thuidium kanedae* | 1 | 1 | 1 | 1 |
| *Thuidium plumulosum* | 0 | 0 | 0 | 1 |
| *Thuidium pristocalyx* | 1 | 0 | 1 | 1 |
| *Thuidium recognitum* | 0 | 1 | 0 | 0 |
| *Thuidium subglaucinum* | 0 | 0 | 1 | 1 |
| *Thuidium submicropteris* | 1 | 1 | 1 | 1 |
| *Thuidium tamariscinum* | 0 | 1 | 0 | 0 |
| *Timmiella anomala* | 1 | 0 | 0 | 0 |
| *Tortella humilis* | 0 | 0 | 0 | 1 |
| *Tortella tortuosa* | 1 | 0 | 0 | 1 |
| *Tortula hoppeana* | 0 | 0 | 1 | 0 |
| *Trachycladiella aurea* | 1 | 0 | 1 | 0 |
| *Trachycystis microphylla* | 1 | 1 | 1 | 1 |
| *Trachycystis ussuriensis* | 0 | 0 | 0 | 1 |
| *Trachyphyllum inflexum* | 0 | 0 | 1 | 0 |
| *Trachypus bicolor* | 1 | 1 | 0 | 1 |
| *Trachypus humilis* | 1 | 0 | 1 | 1 |
| *Trachypus longifolius* | 0 | 0 | 0 | 1 |
| *Trematodon longicollis* | 1 | 0 | 0 | 0 |
| *Trichocolea tomentella* | 1 | 0 | 0 | 0 |
| *Trichocoleopsis sacculata* | 1 | 0 | 0 | 0 |
| *Trichosteleum lutschianum* | 0 | 0 | 0 | 1 |
| *Trichostomum brachydontium* | 1 | 0 | 1 | 1 |
| *Trichostomum crispulum* | 1 | 0 | 1 | 0 |
| *Trichostomum hattorianum* | 0 | 0 | 1 | 1 |
| *Trichostomum planifolium* | 0 | 0 | 1 | 0 |
| *Trichostomum platyphyllum* | 0 | 0 | 0 | 1 |
| *Trichostomum tenuirostre* | 1 | 0 | 0 | 1 |
| *Trichostomum zanderi* | 0 | 0 | 0 | 1 |
| *Venturiella sinensis* | 1 | 0 | 0 | 0 |
| *Vesicularia ferriei* | 0 | 0 | 1 | 1 |
| *Warburgiella cupressinoides* | 0 | 1 | 0 | 1 |
| *Weissia controversa* | 1 | 0 | 1 | 1 |
| *Weissia edentula* | 1 | 1 | 1 | 0 |
| *Wijkia deflexifolia* | 1 | 0 | 1 | 1 |
| *Wijkia hornschuchii* | 0 | 0 | 1 | 0 |
| *Wijkia surcularis* | 0 | 0 | 0 | 1 |
| *Calypogeia integristipula* | 0 | 0 | 0 | 1 |
| *Calyptrochaeta japonica* | 0 | 0 | 0 | 1 |
| *Campylopus flexuosus* | 0 | 0 | 0 | 1 |
| *Dicranella amplexans* | 0 | 0 | 0 | 1 |
| *Floribundaria setschwanica* | 0 | 0 | 0 | 1 |
| *Floribundaria walkeri* | 0 | 0 | 0 | 1 |
| *Heterophyllium affine* | 0 | 0 | 0 | 1 |
| *Plagiothecium euryphyllum* var. *brevirameum* | 0 | 0 | 0 | 1 |
| *Plagiothecium formosicum* var. *rectiapex* | 0 | 0 | 0 | 1 |
| *Plagiothecium laetum* | 0 | 0 | 0 | 1 |
| *Regmatodon longinervis* | 0 | 0 | 0 | 1 |
| *Sematophyllum phoeniceum* | 0 | 0 | 0 | 1 |
| *Ulota crispa* | 0 | 0 | 0 | 1 |
| *Ulota gymnostoma* | 0 | 0 | 0 | 1 |
| *Vesicularia reticulata* | 0 | 0 | 0 | 1 |
| *Weissia breviseta* | 0 | 0 | 0 | 1 |
| *Frullania diversitexta* | 0 | 0 | 0 | 1 |
| *Lejeunea discreta* | 0 | 0 | 0 | 1 |
| *Lejeunea pallidevirens* | 0 | 0 | 0 | 1 |
| *Metzgeria lindbergii* | 0 | 0 | 0 | 1 |
| *Porella oblongifolia* | 0 | 0 | 0 | 1 |
| *Radula oyamensis* | 0 | 0 | 0 | 1 |
